# Author Correction: HLA allele-calling using multi-ancestry whole-exome sequencing from the UK Biobank identifies 129 novel associations in 11 autoimmune diseases

**DOI:** 10.1038/s42003-026-09877-4

**Published:** 2026-04-02

**Authors:** Guillaume Butler-Laporte, Joseph Farjoun, Tomoko Nakanishi, Tianyuan Lu, Erik Abner, Yiheng Chen, Michael Hultström, Andres Metspalu, Lili Milani, Reedik Mägi, Mari Nelis, Georgi Hudjashov, Estonian Biobank Research Team, Erik Abner, Andres Metspalu, Lili Milani, Reedik Mägi, Mari Nelis, Georgi Hudjashov, Tõnu Esko, Satoshi Yoshiji, Yann Ilboudo, Kevin Y. H. Liang, Chen-Yang Su, Julian D. S. Willett, Tõnu Esko, Sirui Zhou, Vincenzo Forgetta, Daniel Taliun, J. Brent Richards

**Affiliations:** 1 Department of Epidemiology, Biostatistics and Occupational Health, McGill University, Montréal, QC Canada; 2 Lady Davis Institute, Jewish General Hospital, McGill University, Montréal, QC Canada; 3 Wellcome Trust Centre for Human Genetics, University of Oxford, Oxford, UK; 4 Kyoto-McGill International Collaborative School in Genomic Medicine, Graduate School of Medicine, Kyoto University, Kyoto, Japan; 5 Department of Human Genetics, McGill University, Montréal, QC Canada; 6 Research Fellow, Japan Society for the Promotion of Science, Tokyo, Japan; 7 5 Prime Sciences Inc, Montreal, Quebec, Canada; 8 Estonian Genome Center, Institute of Genomics, University of Tartu, Tartu, Estonia; 9 Integrative Physiology, Department of Medical Cell Biology, Uppsala University, Uppsala, Sweden; 10 Anaesthesiology and Intensive Care Medicine, Department of Surgical Sciences, Uppsala University, Uppsala, Sweden; 11 Department of Twin Research, King’s College London, London, UK; 12 Infectious Diseases and Immunity in Global Health Program, Research Institute of the McGill University Health Centre, Montréal, Québec, Canada

**Keywords:** Immunogenetics, Sequencing

Correction to: *Communications Biology* 10.1038/s42003-023-05496-5, published online 03 November 2023

In this article the author name ‘Julian D. S. Willett’ was incorrectly written as ‘Julian D. S. Willet’. The original article has been corrected.

