# Supplementary material for: HLA allele-calling using multi-ancestry whole-exome sequencing from the UK Biobank identifies 129 novel associations in 11 autoimmune diseases
Source: Commun Biol. 2023 Nov 3;6:1113. doi: 10.1038/s42003-023-05496-5 (PMC10624861; doi:10.1038/s42003-023-05496-5)
Supplement: Supplementary file 2 — Supplementary Information [file 42003_2023_5496_MOESM2_ESM.pdf]

Supplementary Figure 1: number of reads for each gene

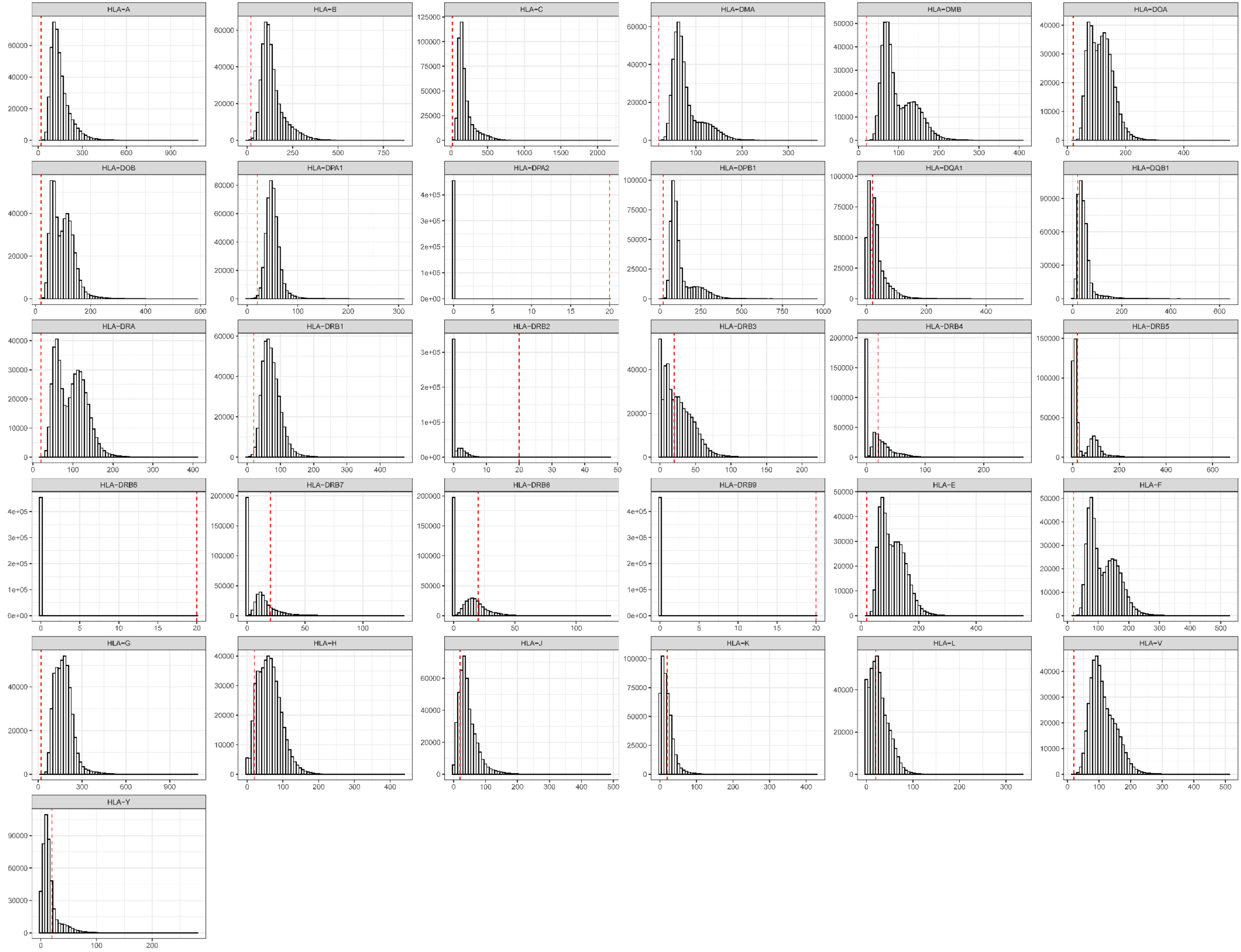

Histograms of the total number of reads for each gene for each participant. Vertical dashed line at 20.

Supplementary Figure 2: HLA allele distribution for common alleles

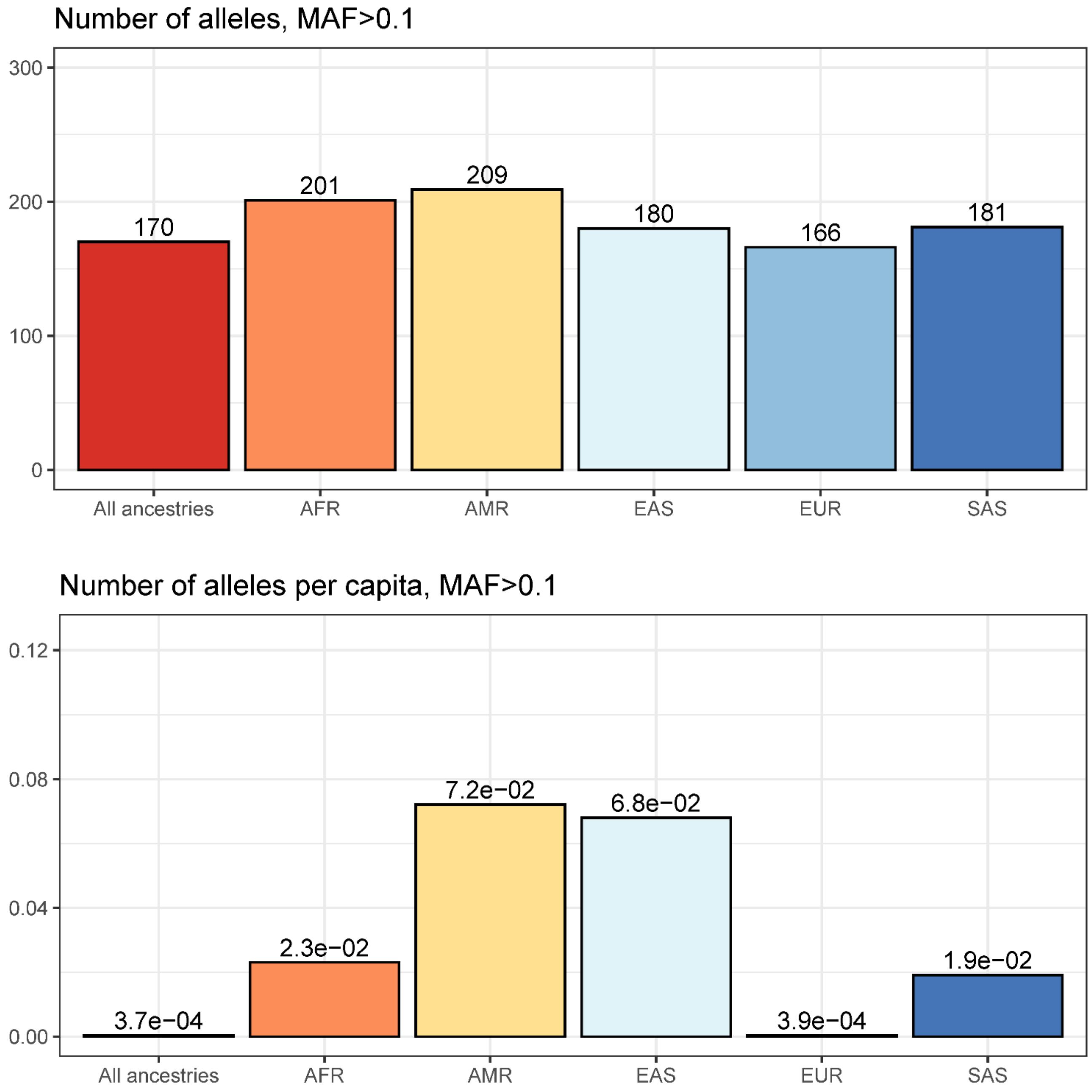

Number of 2-field HLA alleles of minor allele frequency (MAF) > 0.1% per continental genetic ancestry. The analysis is limited to the protein-coding genes.

Supplementary Figure 3: HLA allele down sampling analysis.

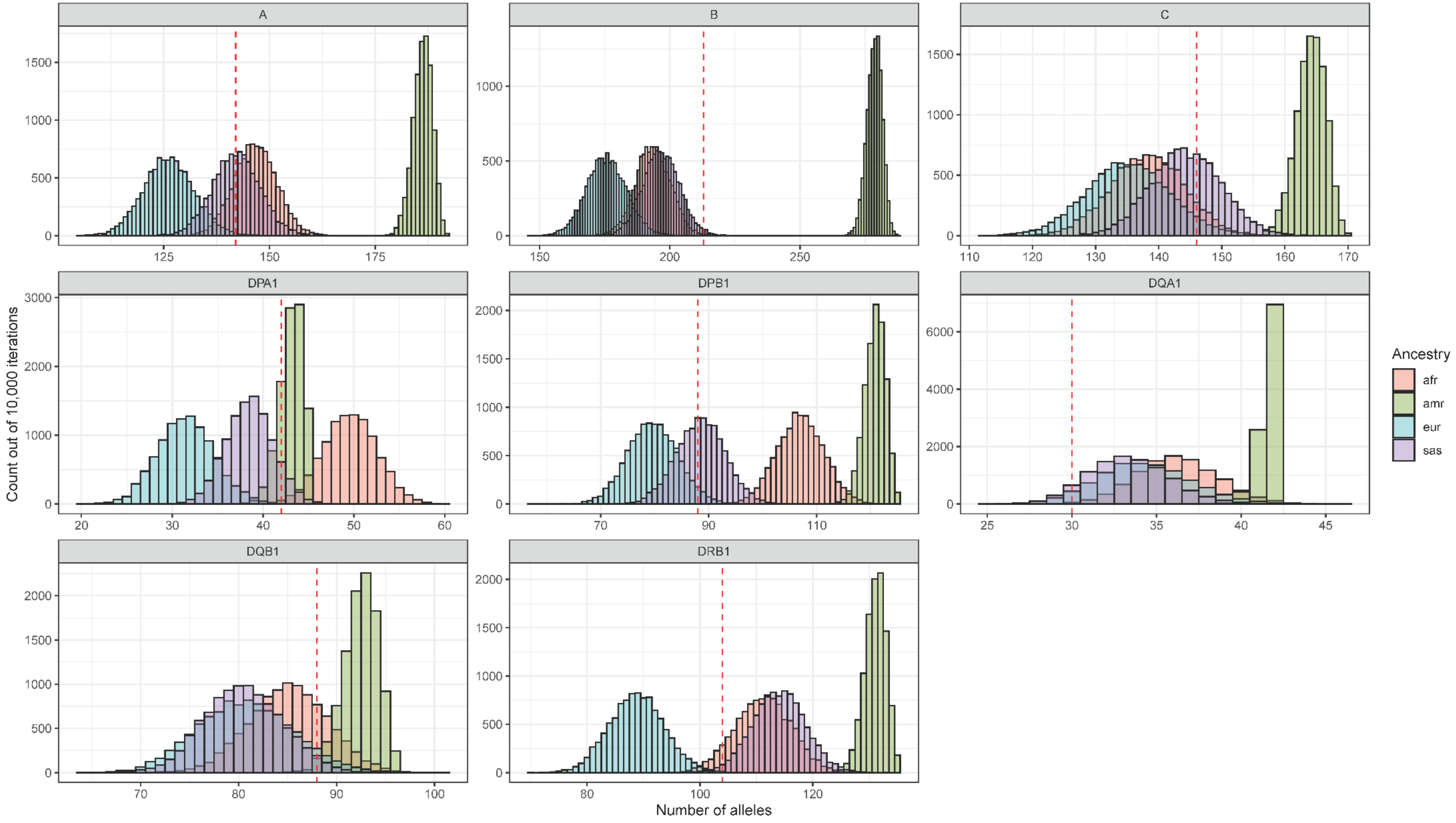

To better assess HLA allele distribution in each ancestry, adjusting for large sample size bias, each ancestry's alleles were resampled without replacement for a total number of alleles equal to the one found in the EAS ancestry (the one with the smallest number of participants). This was done 10,000 times. Histograms refer to the number of unique alleles found in each iteration. The AMR population consistently shows greater HLA variability then other ancestries (except at *HLA-DPA1*). The red vertical dashed bar represents the EAS ancestry (which was not down sampled). See supplementary table **Supplementary Data 28** for full summary statistics of this analysis.

Supplementary Figure 4: Association between concordance and allele frequency.

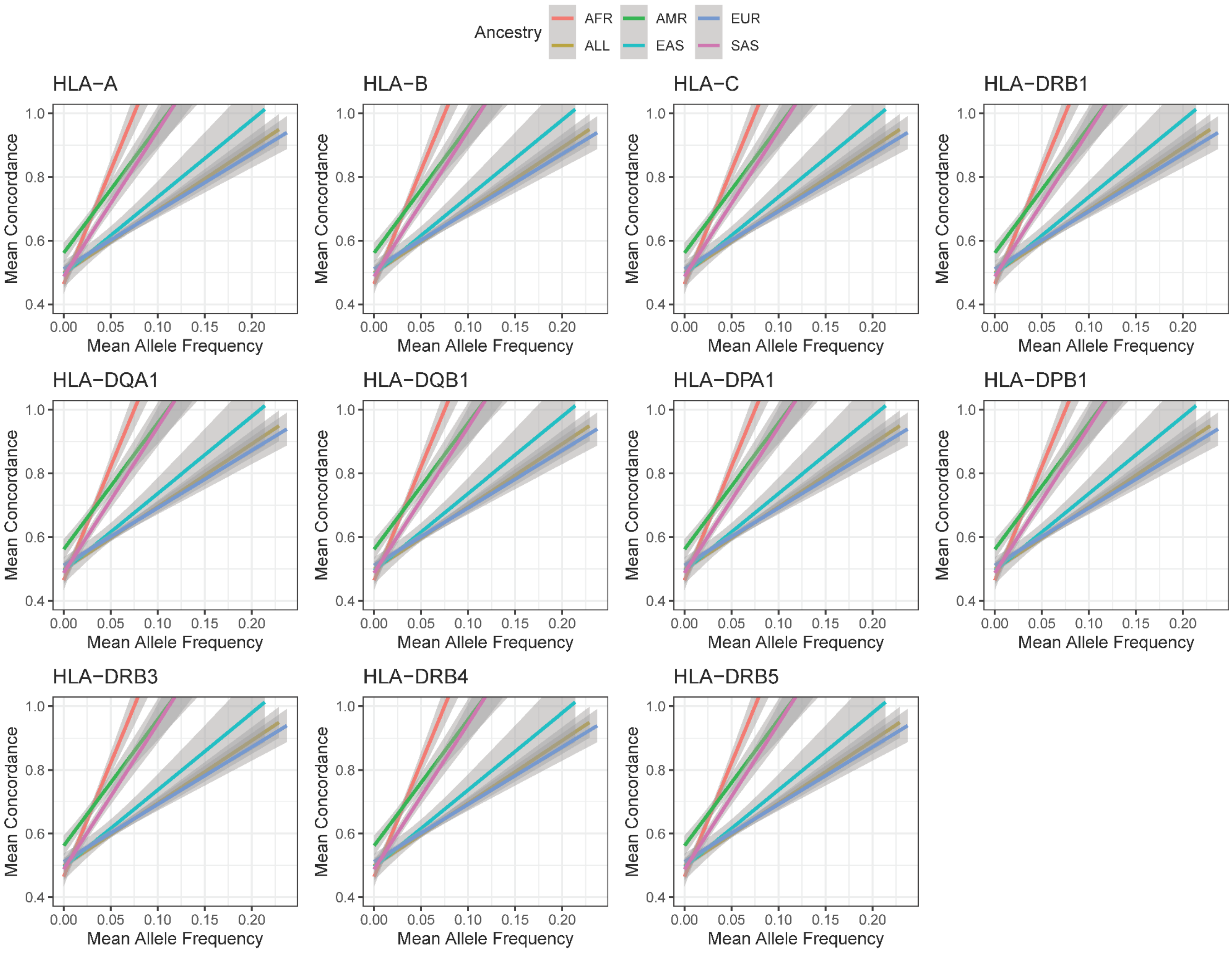

Association (and 95% confidence interval) between concordance and allele frequency.

Supplementary Figure 5: LD between genes for each ancestry.

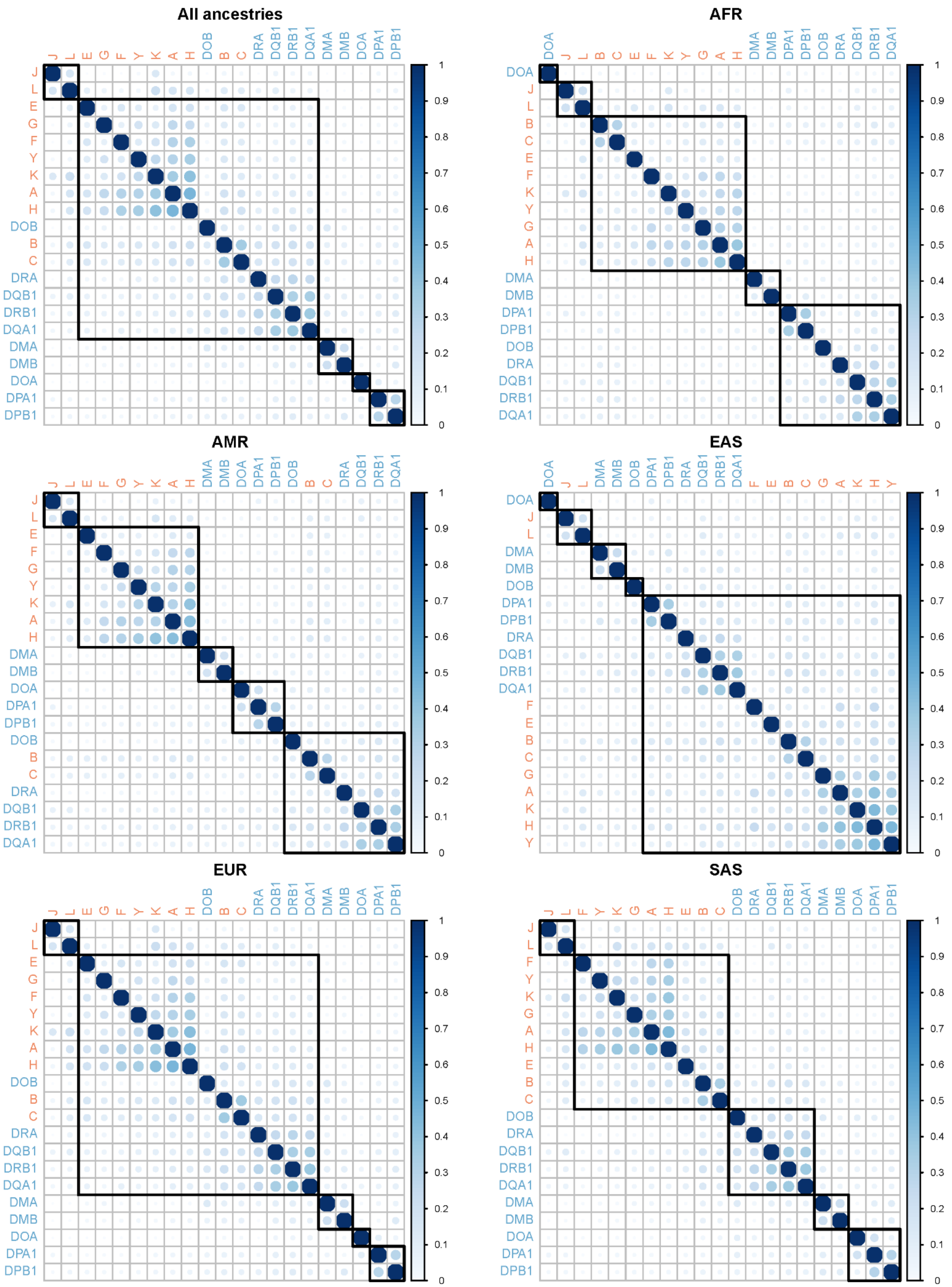

Mean asymmetric LD per ancestry and genes. Choices of squares were drawn using hierarchical clustering. Red: MHC Class I genes. Blue: MHC Class II genes.

Supplementary Figure 6: allele frequency concordance with the AFND reference cohorts

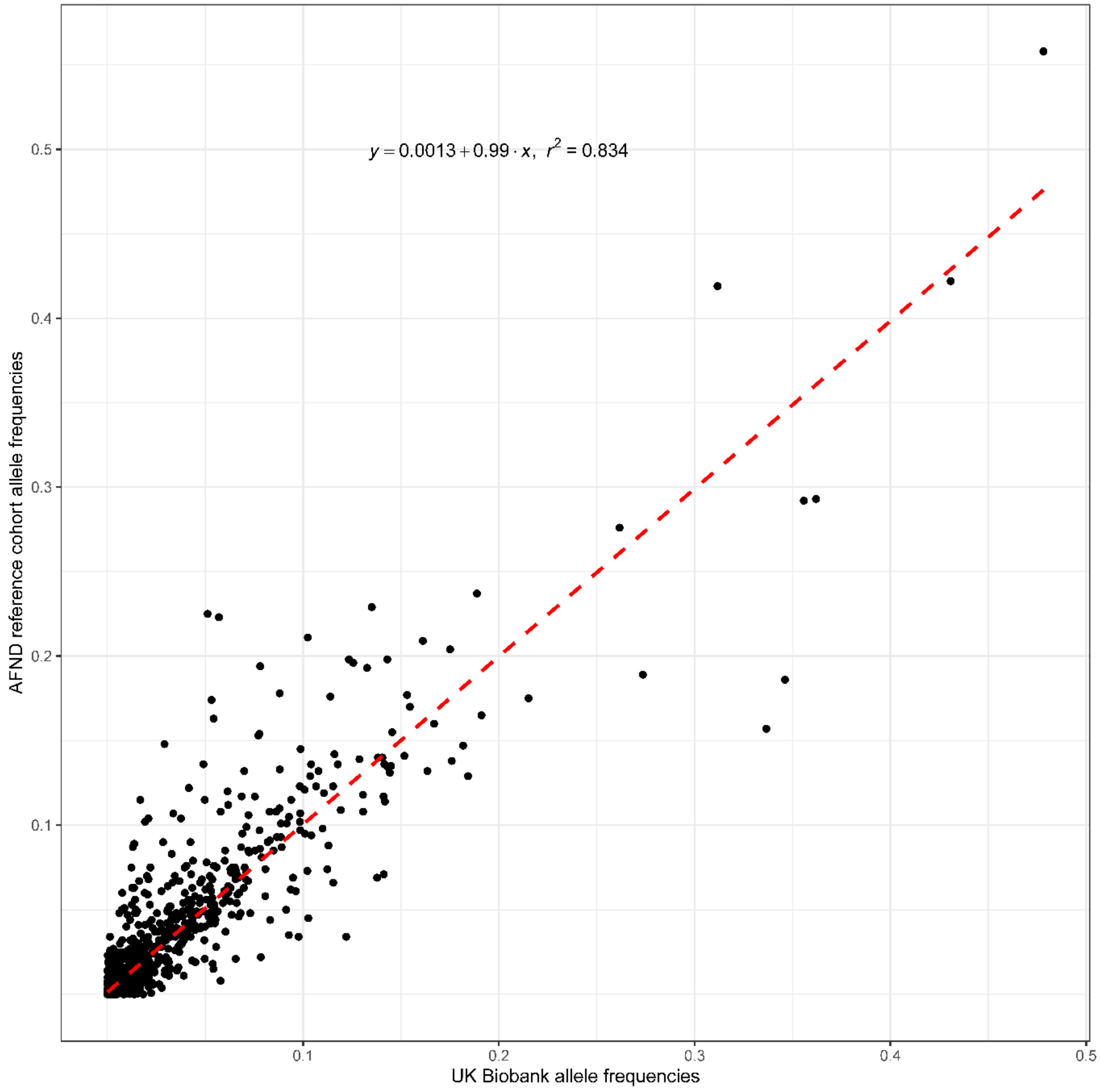

The correlation between UKB WES HLA calling allele frequencies (x-axis) and the selected AFND reference cohorts (y-axis). Comparisons are made separately for each genetic ancestry. The dashed red line shows perfect concordance

Supplementary Figure 7: HLA association studies at 3-field resolution.

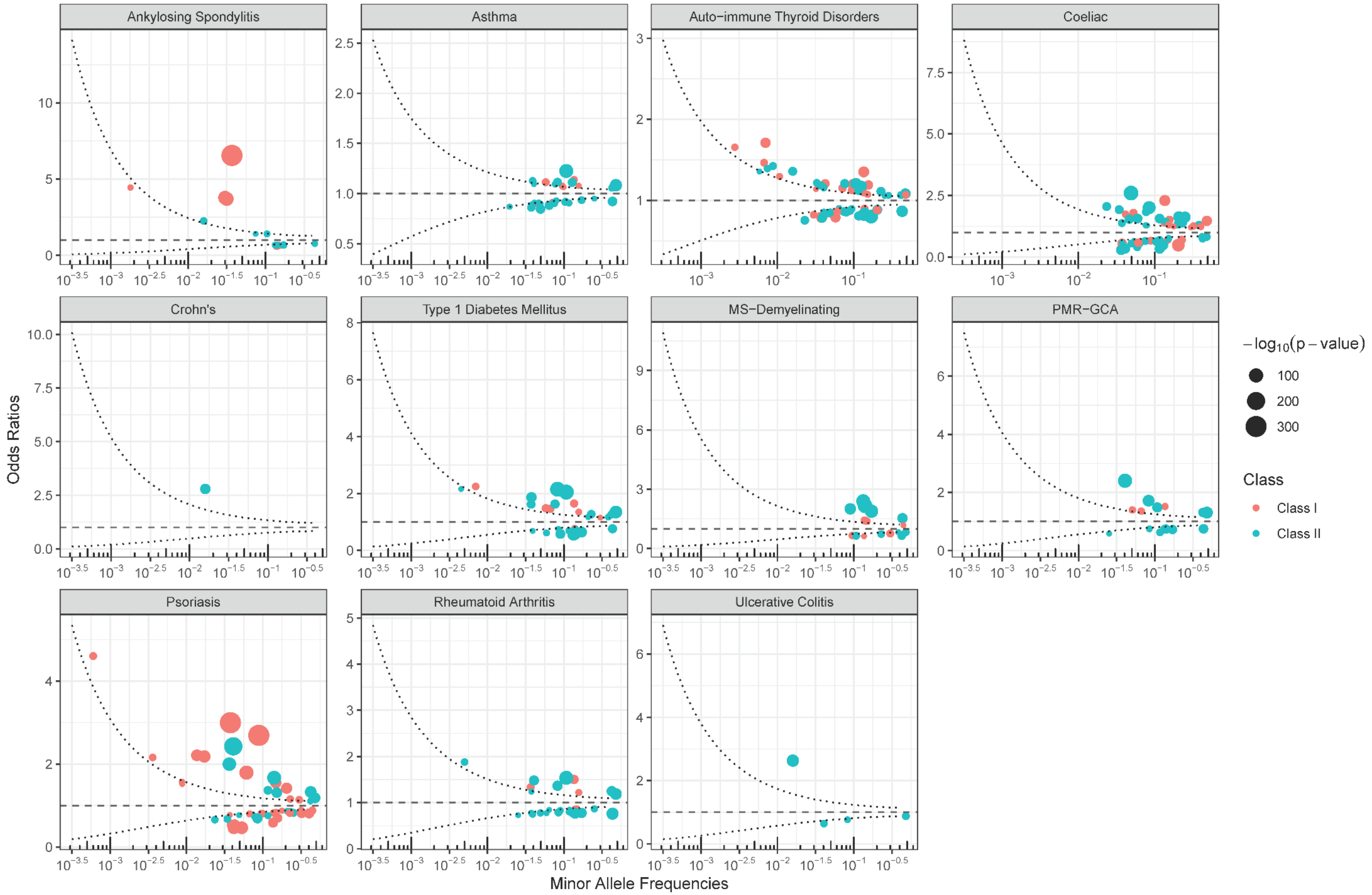

Odds ratios are shown. The curved dashed lines represent a power of 80% to detect an association at a p-value of $(5x10^{-8})/11$ or less.

Supplementary Figure 8: HLA allele associations heterogeneity tests

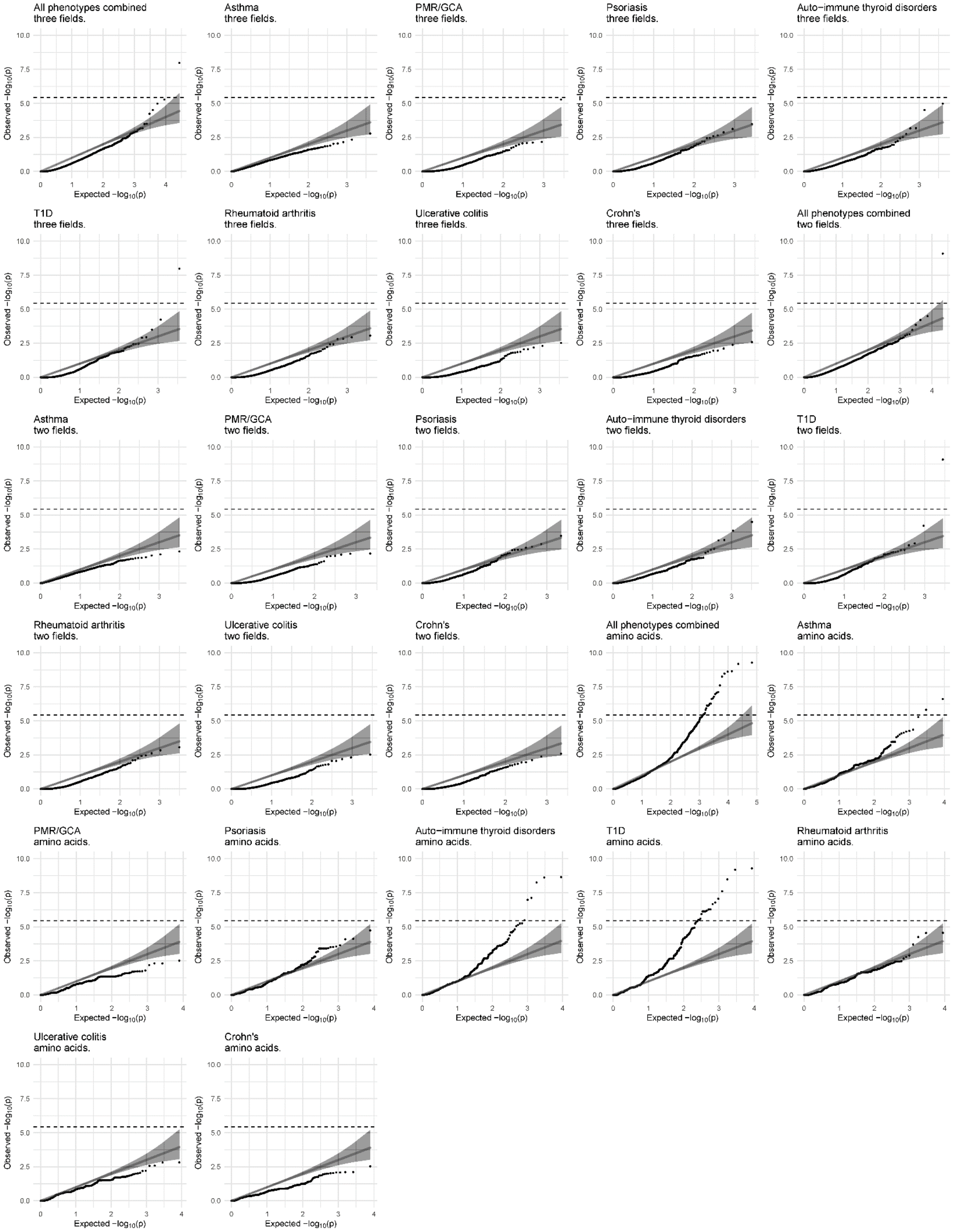

qq-Plots of heterogeneity of association tests across ancestries at a three-field resolution. The vertical dashed line represents the Bonferroni adjusted threshold for this analysis (p = 0.05/13,576). The only HLA allele showing some evidence of heterogeneous association is *DOB*01:02:01* for type 1 diabetes mellitus (T1D). For amino acids, multiple residues at *DOB*, *DRB1*, *DRB5*, and *DQB1* contributed to the signal for type 1 diabetes mellitus and auto-immune thyroid disorders. Grey bars represent 95% confidence intervals expected from fully random results. Full summary statistics can be found in supplementary table **Supplementary Data 20**.

Supplementary Figure 9: Canonical correlation analysis results.

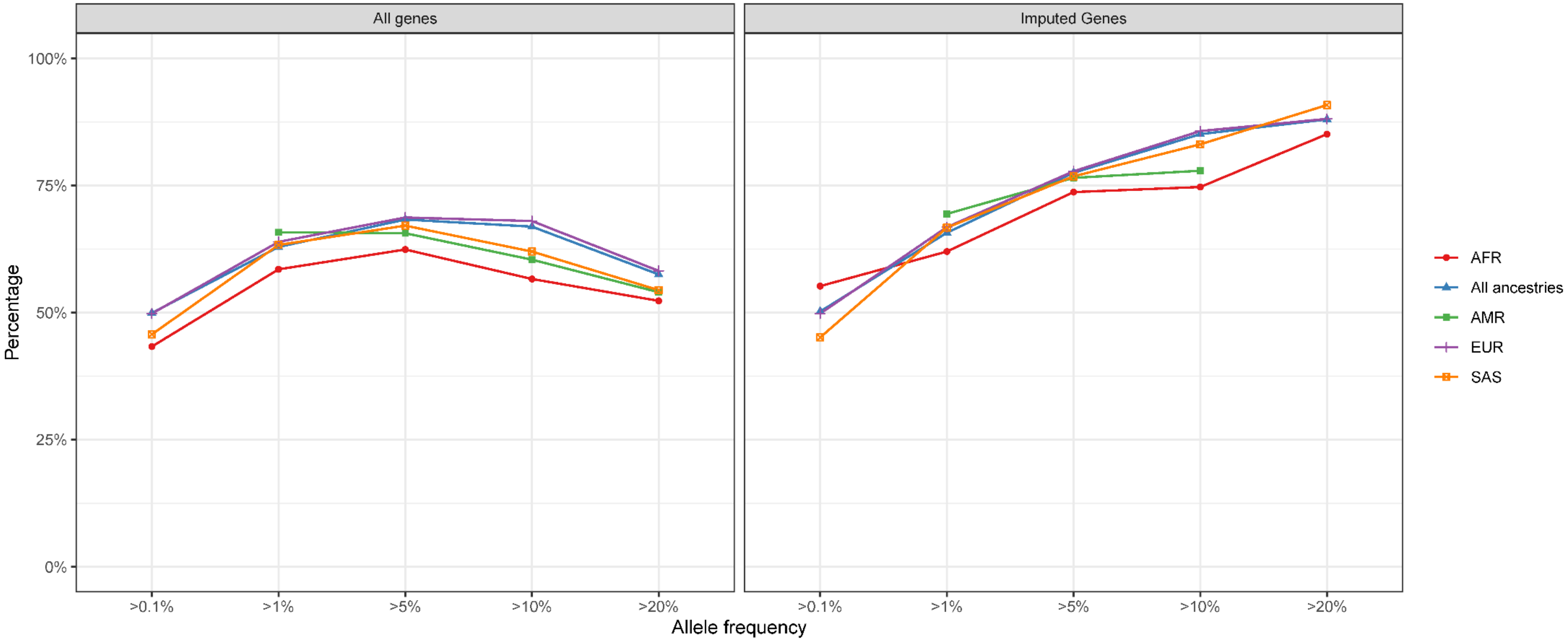

The y-axis refers to the percentage of variance in WES HLA called alleles explained by imputed alleles. The x-axis is the allele frequency threshold in the WES HLA called alleles used for the analysis. Note that due to smaller sample sizes in certain genetic ancestries, CCA calculations were not always stable enough to be performed (e.g. the analysis was not done for AMR genetic ancestry at all). Also, refer to supplementary table **Supplementary Data 25**.

Supplementary Figure 10: Precision-recall curves for each PRS.

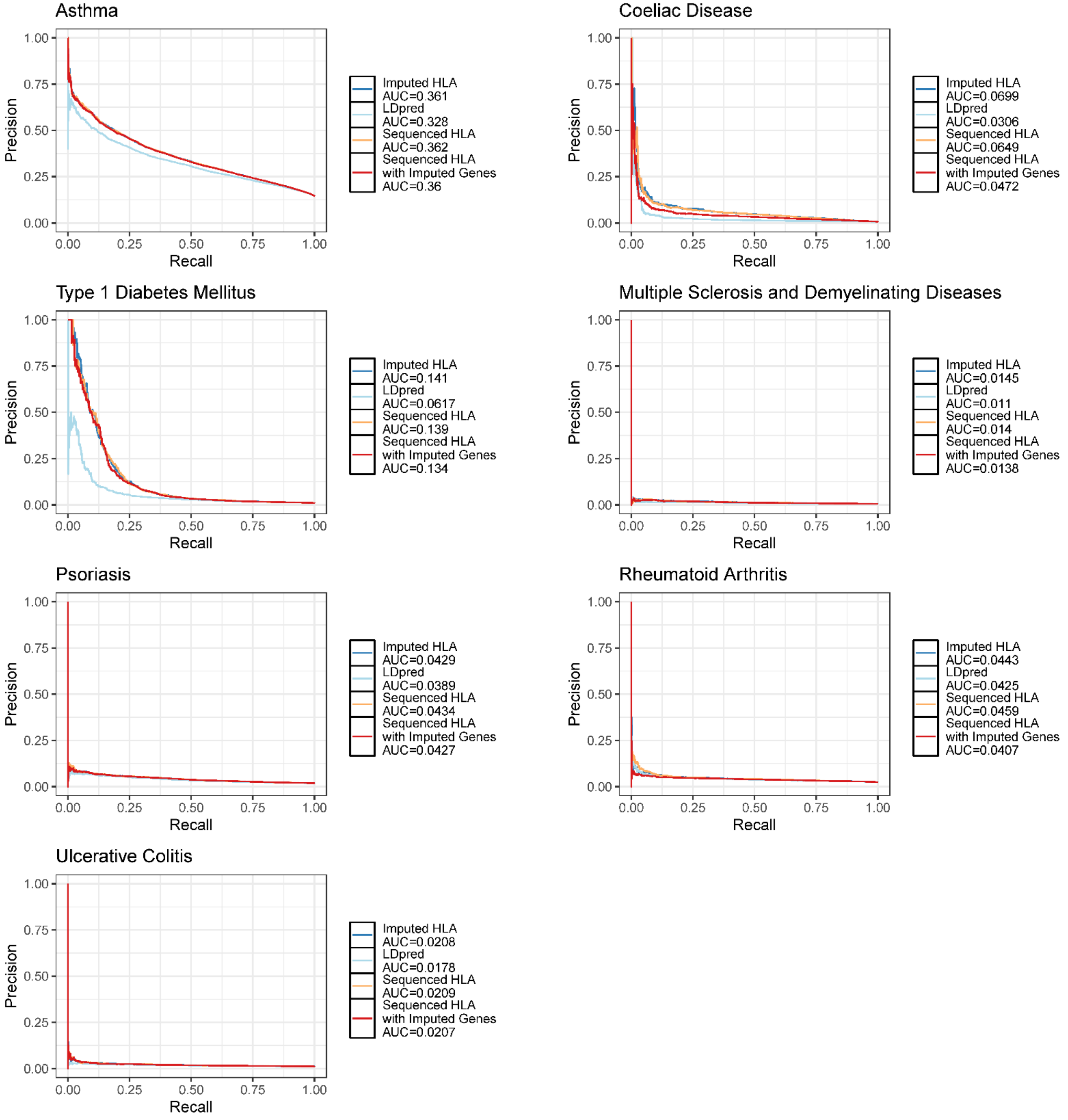

See main text for full explanation of the different PRS. AUC: area under the curve.

Supplementary Figure 11: Negative control phenotypes

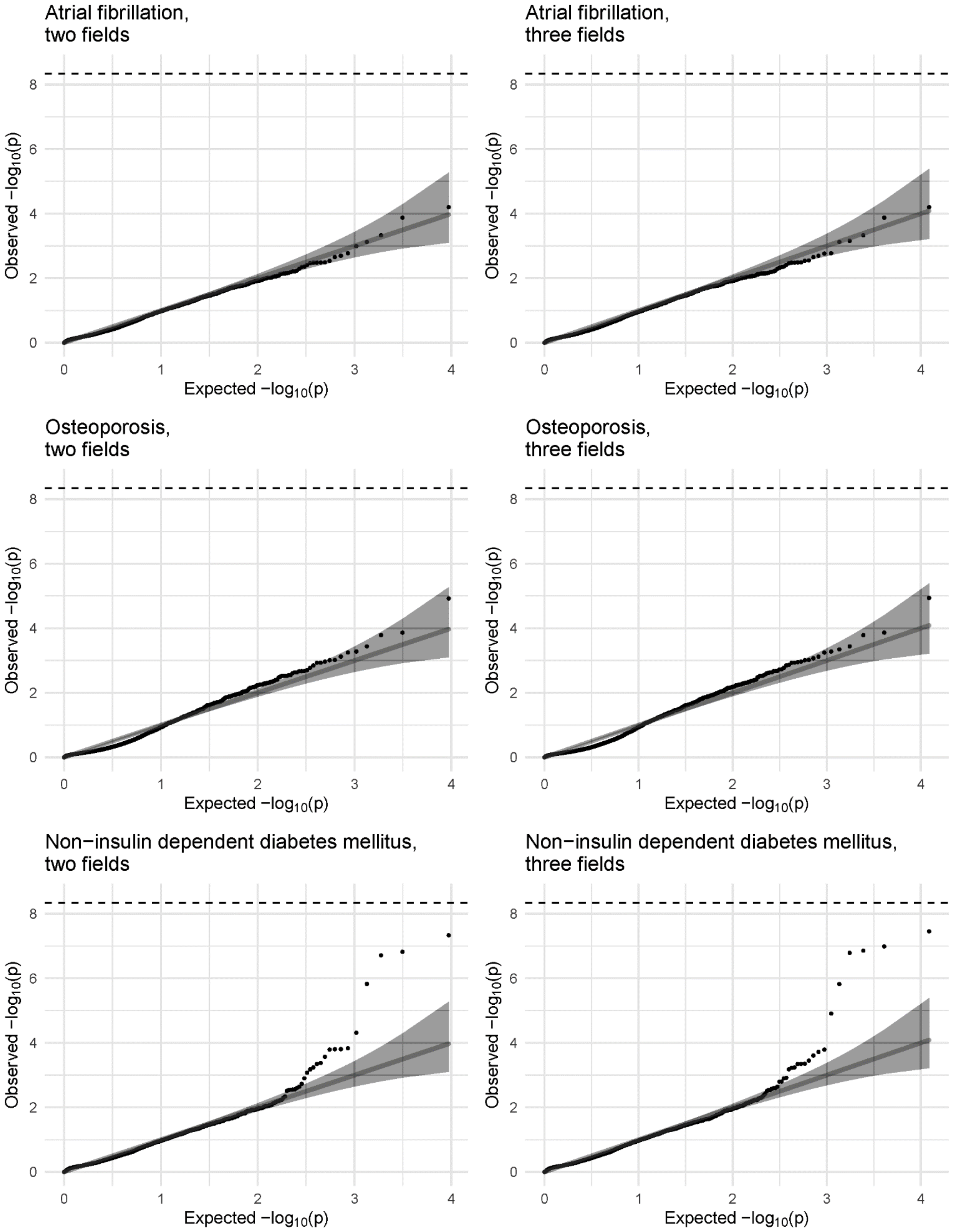

Negative control phenotypes qq-Plots. Horizontal dashed bars represent the p-value threshold for a significant association. Grey bars represent 95% confidence intervals expected from fully random results.
