# Supplementary material for: HLA allele-calling using multi-ancestry whole-exome sequencing from the UK Biobank identifies 129 novel associations in 11 autoimmune diseases
Source: Commun Biol. 2023 Nov 3;6:1113. doi: 10.1038/s42003-023-05496-5 (PMC10624861; doi:10.1038/s42003-023-05496-5)
Supplement: Supplementary file 3 — Description of Additional Supplementary Files [file 42003_2023_5496_MOESM3_ESM.pdf]

Description of Additional Supplementary Files

File name: Supplementary Data 1
Description: HLA gene read coverage.

File name: Supplementary Data 2
Description: HLA allele down sampling results.

File name: Supplementary Data 3
Description: HLA allele frequencies (3-field).

File name: Supplementary Data 4
Description: HLA allele frequencies (2-field).

File name: Supplementary Data 5
Description: Mean allele frequencies of mismatched alleles between our HLA calls and the UK Biobank imputed alleles.

File name: Supplementary Data 6
Description: Full breakdown of imputed and sequenced alleles comparisons (all ancestries combined).

File name: Supplementary Data 7
Description: Full breakdown of imputed and sequenced alleles comparisons (AFR ancestry).

File name: Supplementary Data 8
Description: Full breakdown of imputed and sequenced alleles comparisons (AMR ancestry).

File name: Supplementary Data 9
Description: Full breakdown of imputed and sequenced alleles comparisons (EAS ancestry).

File name: Supplementary Data 10
Description: Full breakdown of imputed and sequenced alleles comparisons (EUR ancestry).

File name: Supplementary Data 11
Description: Full breakdown of imputed and sequenced alleles comparisons (SAS ancestry).

File name: Supplementary Data 12
Description: Asymmetric multiallelic LD between HLA alleles (all ancestries combined).

File name: Supplementary Data 13
Description: Asymmetric multiallelic LD between HLA alleles (AFR ancestry).

File name: Supplementary Data 14
Description: Asymmetric multiallelic LD between HLA alleles (AMR ancestry).

File name: Supplementary Data 15
Description: Asymmetric multiallelic LD between HLA alleles (EAS ancestry).

File name: Supplementary Data 16
Description: Asymmetric multiallelic LD between HLA alleles (EUR ancestry).

File name: Supplementary Data 17
Description: Asymmetric multiallelic LD between HLA alleles (SAS ancestry).

File name: Supplementary Data 18
Description: Codes used to assign each phenotype to participants.

File name: Supplementary Data 19
Description: Phenotypes analyzed for each genetic ancestry.

File name: Supplementary Data 20
Description: Full summary statistics.

File name: Supplementary Data 21
Description: Biallelic LD for the significant variants (only pairs of variants with R2 > 0.2 are shown).

File name: Supplementary Data 22
Description: Conditional analyses.

File name: Supplementary Data 23
Description: Potentially novel alleles and comparisons with the Estonian Biobank imputed results.

File name: Supplementary Data 24
Description: Summary statistics of synonymous variants.

File name: Supplementary Data 25
Description: Canonical correlation analysis between imputed and sequenced alleles.

File name: Supplementary Data 26
Description: Summary of polygenic scores AUC

File name: Supplementary Data 27
Description: AFND cohorts used for reference allele frequencies.

File name: Supplementary Data 28
Description: Negative control phenotypes summary statistics.
