# Supplementary material for: HLA allele-calling using multi-ancestry whole-exome sequencing from the UK Biobank identifies 129 novel associations in 11 autoimmune diseases
Source: Commun Biol. 2023 Nov 3;6:1113. doi: 10.1038/s42003-023-05496-5 (PMC10624861; doi:10.1038/s42003-023-05496-5)
Supplement: Supplementary file 5 — Reporting Summary [file 42003_2023_5496_MOESM5_ESM.pdf]

nature portfolio

Corresponding author(s): Guillaume Butler-Laporte

Last updated by author(s): Oct 12, 2023

Reporting Summary

Nature Portfolio wishes to improve the reproducibility of the work that we publish. This form provides structure for consistency and transparency in reporting. For further information on Nature Portfolio policies, see our Editorial Policies and the Editorial Policy Checklist.

Statistics

For all statistical analyses, confirm that the following items are present in the figure legend, table legend, main text, or Methods section.

n/a	Confirmed	
☐	☒	The exact sample size (*n*) for each experimental group/condition, given as a discrete number and unit of measurement
☐	☒	A statement on whether measurements were taken from distinct samples or whether the same sample was measured repeatedly
☐	☒	The statistical test(s) used AND whether they are one- or two-sided<br>*Only common tests should be described solely by name; describe more complex techniques in the Methods section.*
☐	☒	A description of all covariates tested
☐	☒	A description of any assumptions or corrections, such as tests of normality and adjustment for multiple comparisons
☐	☒	A full description of the statistical parameters including central tendency (e.g. means) or other basic estimates (e.g. regression coefficient) AND variation (e.g. standard deviation) or associated estimates of uncertainty (e.g. confidence intervals)
☐	☒	For null hypothesis testing, the test statistic (e.g. *F*, *t*, *r*) with confidence intervals, effect sizes, degrees of freedom and *P* value noted<br>*Give P values as exact values whenever suitable.*
☒	☐	For Bayesian analysis, information on the choice of priors and Markov chain Monte Carlo settings
☒	☐	For hierarchical and complex designs, identification of the appropriate level for tests and full reporting of outcomes
☒	☐	Estimates of effect sizes (e.g. Cohen's *d*, Pearson's *r*), indicating how they were calculated

Our web collection on statistics for biologists contains articles on many of the points above.

Software and code

Policy information about availability of computer code

Data collection	The primary data was collected by the UK Biobank and the Estonian Biobank (for replication analyses).
Data analysis	All code used for the UK Biobank analysis is available on github: https://github.com/DrGBL/HLA_UKB

For manuscripts utilizing custom algorithms or software that are central to the research but not yet described in published literature, software must be made available to editors and reviewers. We strongly encourage code deposition in a community repository (e.g. GitHub). See the Nature Portfolio guidelines for submitting code & software for further information.

Data

Policy information about availability of data

All manuscripts must include a data availability statement. This statement should provide the following information, where applicable:

- Accession codes, unique identifiers, or web links for publicly available datasets
- A description of any restrictions on data availability
- For clinical datasets or third party data, please ensure that the statement adheres to our policy

All code is available on https://github.com/DrGBL/HLA_UKB. The primary data used for all analyses (i.e. WES CRAM files) is available through the UK Biobank DNAnexus research analysis platform. All summary statistics can be found in the supplements.

Research involving human participants, their data, or biological material

Policy information about studies with human participants or human data. See also policy information about sex, gender (identity/presentation), and sexual orientation and race, ethnicity and racism.

Reporting on sex and gender	We used self-reported sex as a covariate in our analyses.
Reporting on race, ethnicity, or other socially relevant groupings	The only groupings used were self-reported sex and genetic ancestry, the latter was only used for the UK Biobank. Genetic ancestry is defined in greater detail in the text, but briefly, each UK Biobank participant's genetic data was projected onto the 1000 Genome reference to determine which genetic ancestry group they most closely matched. Analyses were then performed for each genetic ancestry separately in order to mitigate population stratification bias.
Population characteristics	The UK Biobank population characteristic is well described in many publications, see here for example: https://rdcu.be/deFAe . Similarly for the Estonian Biobank: 10.1093/ije/dyt268
Recruitment	All study participants were recruited by the UK Biobank or the Estonian Biobank (for the replication).
Ethics oversight	The UKB was approved by the Northwest Multi-centre Research Ethics (approval number: 11/NW/0382). The activities of the EstBB are regulated by the Human Genes Research Act, which was adopted in 2000 specifically for the operations of EstBB. Individual level data analysis in EstBB was carried out under ethical approval 1.1-12/624 from the Estonian Committee on Bioethics and Human Research (Estonian Ministry of Social Affairs), using data according to release application 6-7/GI/8592 from the Estonian Biobank. Informed consent was obtained from all participants.

Note that full information on the approval of the study protocol must also be provided in the manuscript.

Field-specific reporting

Please select the one below that is the best fit for your research. If you are not sure, read the appropriate sections before making your selection.

☒ Life sciences ☐ Behavioural & social sciences ☐ Ecological, evolutionary & environmental sciences

For a reference copy of the document with all sections, see nature.com/documents/nr-reporting-summary-flat.pdf

Life sciences study design

All studies must disclose on these points even when the disclosure is negative.

Sample size	We used all the of the UK Biobank participants which had the necessary genetic data for the analyses (i.e. up to 454,824 participants).
Data exclusions	There were no exclusion criteria other than quality control filters on HLA calling results.
Replication	We used all of the Estionian Biobank that had HLA results (i.e. up to 212,955 participants).
Randomization	There was no randomization. Analyses were adjusted for sex, age, 10 genetic principal components, and stratified by genetic ancestry (see above).
Blinding	Not applicable.

Reporting for specific materials, systems and methods

We require information from authors about some types of materials, experimental systems and methods used in many studies. Here, indicate whether each material, system or method listed is relevant to your study. If you are not sure if a list item applies to your research, read the appropriate section before selecting a response.

Materials & experimental systems

n/a	Involved in the study	
☒	☐	Antibodies
☒	☐	Eukaryotic cell lines
☒	☐	Palaeontology and archaeology
☒	☐	Animals and other organisms
☒	☐	Clinical data
☒	☐	Dual use research of concern
☒	☐	Plants

Methods

n/a	Involved in the study	
☒	☐	ChIP-seq
☒	☐	Flow cytometry
☒	☐	MRI-based neuroimaging
